# Beneficial reward-to-risk action of glucosamine during pathogenesis of osteoarthritis

**DOI:** 10.1186/s40001-015-0176-7

**Published:** 2015-10-31

**Authors:** Yeon-Ho Kang, Sujeong Park, Chihyun Ahn, Jinsoo Song, Dongkyun Kim, Eun-Jung Jin

**Affiliations:** Department of Biological Sciences, College of Natural Sciences, Wonkwang University, Iksan, Chunbuk 570-749 Korea; Integrated Omics Institute, Wonkwang University, Iksan, Chunbuk 570-749 Korea

**Keywords:** Glucosamine, Apoptosis, Autophagy, Pexophagy, Peroxidation, Short time exposure, Long time exposure, Beneficial reward-to-risk action

## Abstract

**Objective:**

Glucosamine is widely used to improve the symptoms and to delay the structural progression of osteoarthritis. However, its efficacy in osteoarthritis has been controversial and its underlying mechanism of action remains unclear. The aim of this study was to investigate the effects of glucosamine and the underlying mechanisms in human chondrocytes.

**Methods:**

Chondrocytes from normal human articular cartilage were treated with glucosamine (10–100 mM). Subsequently, cell death was analyzed by Annexin V staining and FACS and mitochondrial function was studied by measuring the mitopotential. Peroxisomal function was analyzed by BODIPY staining, and gene expression of PMP70 and acyl-CoA oxidase 1, by real-time PCR. Total lipids were analyzed by gas chromatography/mass spectrometry. Autophagy activation was determined by western blotting of beclin and light chain 3B. Autophagosome formation was analyzed by introduction of green fluorescent protein (GFP) LC3, and pexophagy was determined by introduction of mRFP-EGFP-SKL plasmids.

**Results:**

Treatment of chondrocytes with glucosamine exerts exposure time-dependent dual effects on apoptosis/autophagy. Short time exposure of glucosamine to chondrocytes activated autophagy, pexophagy, and peroxidation. On the other hand, long time exposure of glucosamine had opposite effects, namely accumulation of very long chain fatty acids and peroxisomal dysfunction.

**Conclusion:**

We highlight the dual role of glucosamine in apoptosis/autophagy in human chondrocytes depending on exposure time. Although further research is required to fully understand the dual effects of glucosamine, dosage and duration of glucosamine treatment are clear contributing factors towards the line of beneficial reward-to-risk action.

## Background

Osteoarthritis (OA), one of the most disabling arthritic conditions, affects not only cartilage, but also induces metabolic and structural modifications of the subchondral bone and the synovial membrane [1]. Despite the increasing number of OA patients, to date no cure for this disease has been found. Currently, the management of OA consists mostly of symptom management, i.e., the reduction of pain and improvement of joint function with conventional treatment methods such as the use of analgesics or non-steroidal anti-inflammatory drugs [2, 3]. Recently, the failure of these conventional treatments has given rise to the increasing use of symptomatic slow-acting drugs for OA (SYSADOA)-based therapies.

One of the commonly used SYSADOAs is glucosamine [4]. Glucosamine, an amino monosaccharide, is an indispensable component of chondroitin sulfate and keratin sulfate, which are the main glycosaminoglycans (GAG) in the cartilage [5, 6]. Glucosamine has been used in the supplementary treatment of OA, although its effects on the extracellular matrix (ECM) metabolism in chondrocytes remain controversial [7, 8]. In chondrocyte pellet culture, glucosamine acts as a suppressor of interleukin-1 (IL-1)-induced prostaglandin E2 synthase and stimulates proteoglycan synthesis [9–11]. Administration of glucosamine in a chymopapain-induced damaged rabbit joint [12] and murine OA model [13] resulted in increased cartilage matrix. Moreover, the investigators reported mild protection efficacy of glucosamine in human articular cartilage, including the reduction of surface fibrillation, loss of safranin O staining, and loss of sulfated glycosaminoglycans [14]. In 2013, Caramés et al. demonstrated the therapeutic efficacy of glucosamine in OA by providing evidence that it activated autophagy by inhibiting the AKT/FoxO3/mammalian target of the rapamycin pathway [15]. They suggested that a specific form of autophagy, mitophagy, could remove the damaged mitochondria frequently observed in OA cartilage.

On the other hand, in 2014, Jiang et al. highlighted the negative effect of autophagic cell death in articular chondrocytes [16]. They observed that high concentrations of glucosamine had a negative effect on cell viability, possibly due to autophagic cell death.

To date, a dual role for glucosamine has been implied, i.e., a potentially desired chondro-protective effect and an undesired negative effect. Therefore, in studying the effects of glucosamine, particular attention should be paid to the regulatory signals and molecules responsible for these dual functions. Here, we attempt to elucidate the underlying regulatory mechanisms that may determine the switch between these two possible opposing functions.

Although chondrocytes reportedly produce only 25 % of their total ATP via oxidative phosphorylation (OXPHOS) [17], recent studies have demonstrated that, in humans, OA chondrocytes demonstrated lower activity of mitochondrial respiratory chain complexes I, II, and III than normal chondrocytes [18]. In addition, this mitochondrial dysfunction may further affect the pathophysiological features of OA, such as cartilage degradation, via reduced chondrocyte biosynthesis and growth, cartilage matrix calcification, and increased chondrocyte apoptosis and inflammatory responses. Moreover, inflammatory cytokines such as TNF-α and IL-1β decrease ATP levels and mitochondrial membrane potential [19]. Taken together, these reports suggest that mitochondrial damage may play a significant role in the pathogenesis of OA. Recently, we suggested the possible functional interconnection between mitochondria and peroxisome [20], i.e., peroxisomal dysfunction may trigger mitochondrial dysfunction in human articular chondrocytes. However, these functional interconnections during OA pathogenesis remain poorly understood and need to be further characterized. In the present study, we investigated the dual functions of glucosamine in human articular chondrocytes and assessed whether peroxisomal dysfunction may play a role in regulating these glucosamine-induced beneficial and disadvantageous functions.

## Methods

### Cell line and cell culture

Human chondrocytes derived from normal human articular cartilage were purchased from Cell Applications (San Diego, CA). Cells were cultured in chondrocyte growth medium (Cell Applications). Cells were maintained in a humidified incubator at 37 °C with 5 % CO_2_. To check the differential ability of chondrocytes, pellet cultures were applied and stained with Alcian blue and the RNA levels of type II collagen and aggrecan were analyzed before the experiments. For this study, cells were treated with 10, 30, 50 and 100 mM d-(+)-glucosamine hydrochloride for 2 h (short time exposure) or 24 h (long time exposure) unless exposure time is indicated, respectively (Sigma-Aldrich, St. Louis, MO, dissolved in growth media).

### Pellet culture of chondrocytes

For differentiation, alginate beads with encapsulated human chondrocytes (Cell applications, San Diego, CA, USA) were grown in a chondrocyte differentiation medium (Cell applications, San Diego, CA, USA) for 2 weeks. Briefly, human chondrocyte were suspended at a density of 4 × 10^6^ per milliliters in a 1.2 % solution of sterile alginate in 0.15 M NaCl. The cell suspension was slowly expressed through a 22-gauge needle and dropped into a 102-mM CaCl_2_ solution. The beads with approximately 40,000 cells/bead (diameter 3 mm) were allowed to polymerize for 10 min and washed twice with 0.15 M NaCl, followed by two washes in DMEM/F12. The beads were then transferred to medium and cultured at 37 °C in a humidified atmosphere of 5 % CO_2_. Alcian blue staining was used to detect chondrocyte nodule formation after 2 weeks of alginate beads culture. Alginate beads were rinsed with PBS and fixed in 4 % paraformaldehyde in PBS for 20 min. Alginate Beads were washed with PBS three times and stained in 1 % Alcian blue in 0.1 N HCl for 8 h at room temperature. Alginate Beads were de-stained in 0.1 N HCl two times and stored in water for image capture.

### Quantitative real-time PCR (qRT-PCR)

Total RNA was isolated using RNAiso Plus (TaKaRa, Japan) according to the manufacturer’s protocol. Aliquots of total RNA (1 μg) from each sample were reverse-transcribed into cDNA according to the instructions of the PrimeScript 1st strand cDNA Synthesis Kit (TaKaRa, Japan). Quantitative real-time PCR (qRT-PCR) was performed using the StepOnePlus Real-Time PCR System (Applied Biosystems, Foster City, CA, USA). PCR were prepared and heated to 95 °C for 2 min followed by 40 cycles of denaturation at 95 °C for 15 s, annealing at specific Tm for 15 s, and extension at 72 °C for 20 s. Quantification of the PCR signals was achieved by comparing the cycle threshold value (*C*_t_) of the gene of interest with the *C*_t_ value of the reference gene *GAPDH*. The following oligonucleotides were used as primers: *MMP13*: 5′-TTGCAGAGCGCTACCTGAGATCAT-3′, antisense, 5′-TTTGCCAGTCACCT CTAAGCCGAA-3′, sense; *ADAMTS4*: 5′-CGCTTTGCTTCACTGAGTAGAT-3′, antisense, 5′-CTGTTAGCAGGTAGCGCTTTAG-3′, sense; *PMP70*: 5′- CCAGTTGGGTCATATCCT TGAA-3′, antisense, 5′- CTTGCCATCGCC ATTCTTTG-3′, sense; *LC3A*: 5′- ACAGCATGG TGAGTGTGTC-3′, antisense, 5′- GGGAGGCGTAGACCATATAGA-3′, sense; *LC3B*: 5′- GCCTTCTTCCTGTTGGTGAA-3′, antisense, 5′- TGGGAGGCATAGACCATGTA-3′, sense; CASP1: 5′-GGCAGGCCTGGA TGATGA-3′, antisense, 5′-ATACCAAGAACT GCCCAAGTTTG-3′, sense, CASP3: 5′-GCGCCCTGGCAGCAT-3′, antisense, 5′-GCCTACAGCCCATTTCTCCAT-3′ sense, CASP9: 5′-AACAGCATTAGCGACCCTAAGC-3′ antisense, 5′-AGCAGTGGGCTCAC TCTGAAG-3′, sense, FAS: 5′-CCAGCATGG TTGTTGAGCAA-3′ antisense, 5′-ACCCGCTCAGTACGGAGTTG-3′, sense, COMP: 5′-TCACAAGCATCTCCCACAAA-3′ antisense, 5′-GACAGTGATGGCGATGGTATAG-3′ sense, ACAN: 5′-TCGAGGGTGT AGCGTGTAGAGA-3′ antisense, 5′-TCGAGGACA GCGAGGCC-3′ sense, *GAPDH:* 5′-GATCATCAGCAATGCCTCCT-3′, antisense, 5′-TGTGGTCATG AGTCCT TCCA-3′, sense.

### Annexin V and viability cell assays

The apoptotic and necrotic cell populations were analyzed by Muse Cell Analyzer (Merck Millipore), which is a miniaturized fluorescent flow cytometer. Analyses were performed using specific fluorescent dyes Muse Annexin V and Dead Cell Kit (Merck Millipore). Briefly, both floating and adherent treated cells were collected, centrifuged at 300×*g* for 5 min, and suspended in phosphate buffered saline (PBS). Aliquots of 100 μL of cell suspension were added to 100 μL of diluted Muse Annexin V and Dead Cell reagent and incubated for 20 min at room temperature (RT). Subsequently, cells were analyzed and the percentage of early or late apoptotic cells was determined in accordance with the Millipore guidelines.

### Mitochondrial membrane potential assay

The mitochondrial depolarization state of cells was analyzed by Muse Cell Analyzer (Merck Millipore). We simultaneously measured changes in the mitochondrial membrane potential by assay kit (Merck Millipore). Briefly, both floating and adherent treated cells were collected, centrifuged at 300×*g* for 5 min, and suspended in cell culture medium. Briefly, 100 μL aliquots of cell suspension were first added to 95 μL of diluted Muse Mitopotential dye and mixed for 20 min at 37 °C before incubating with 5 μL of 7-AAD reagent dye at RT. After 5 min, the cell suspensions were analyzed and the percentages of live, depolarized, and dead cells were determined in accordance with the Millipore guidelines.

### BODIPY staining

BODIPY 493/503 or BODIPY 665/676 (Molecular Probes, Carlsbad, CA, USA), was diluted in PBS at a concentration of 1 mg/mL. Following fixation with 4 % paraformaldehyde (PFA) for 10 min and staining with 4,6-diamidino-2-phenylindole (DAPI) for identification of nuclei, the samples were washed 3 times in PBS for 10 min and stained with BODIPY. The samples were mounted in VECTASHIELD (Vector Laboratories), covered with glass cover slips, and digital images were obtained with an Olympus Fluoview FV100 confocal laser scanning microscope under epifluorescent optics.

### Tandem fluorochrome pexophagy assay

Human chondrocytes were transfected with the mRFP-EGFP-SKL plasmid. Two days after the first transfection, the cells were cultured for another 24 h in the presence of lysosomal inhibitors, 120 μM leupeptin (Sigma-Aldrich), and 2 μM E-64 (Enzo Life Sciences). After incubation, the cells were washed in PBS and fixed with 4 % PFA in PBS for 15 min at RT.

### LC3-positive vesicle formation

Human chondrocytes were transfected with LC3-GFP plasmid. Transfection efficiency of the plasmid was assessed visually (>60 % and identical for all wells). After 2 days, the cells were starved for 3 h in the presence of 20 μM chloroquine (InvivoGen) and permeabilized with 0.025 % digitonin in PBS for 5 min to wash out the cytosolic LC3 protein and enrich for the vesicle-associated LC3. Subsequently, the cells were washed in PBS and fixed with 4 % PFA in PBS for 15 min at RT.

### Gas chromatography/mass spectrometry

Total lipids were extracted using a chloroform/methanol (2:1 v/v) mixture. The extracted lipids were separated on a Sep-Pak Silica Cartilage column (Waters, Milford, MA, USA) and transmethylated with 0.5 M CH3ONa in methanol by heating in a sealed tube at 70 °C for 1 h under nitrogen. The fatty acid methyl esters were extracted with hexane. Subsequent gas chromatography–mass spectrometry (GC–mass) analysis was performed according to the standard protocol. Briefly, the sample was injected into a Finnigan MAT-8430 mass spectrometer connected to an HP-5890 gas chromatography (Finnigan MAT, Bremen, Germany) equipped with a DB-5 capillary column.

### Apoptosis and autophagy profiling PCR array

Apoptosis and autophagy profiling was conducted using the RT^2^ Profiler PCR Array Kit (QIAGEN, GmbH, Hilden, Germany), which included specific primers for apoptosis (PAHS-012Z) or autophagy (PAHS-084Z). Each profiling array was performed according to the manufacturer’s instructions. cDNA was synthesized with the RT^2^ First Strand Kit (Qiagen, #330401) and qRT-PCR was performed using the RT^2^ qPCR SYBR Green Fluor qPCR Master Mix (Qiagen, #330512) according to the manufacturer’s instructions. Data were analyzed and gene expression data visualized using GenEx (Weihenstephan, Germany).

### Western Blot

Cells were lysed in RIPA buffer (50 mM Tris–HCl, pH 7.4, containing 150 mM NaCl, 1 % Nonidet P-40, 0.1 % SDS, 0.1 % deoxycholic acid, 10 mM NaF, 10 mM Na_4_P_2_O_7_, 0.4 mM Na_3_VO_4_, and protease inhibitors) for 30 min on ice. The total protein content of the cells was determined by BCA Protein Acid Reagent (bicinchoninic acid) (Pierce Biotechnology Inc., Rockford, MN, USA). Proteins (30 μg) were separated by 10 % polyacrylamide gel electrophoresis containing 0.1 % SDS and transferred to nitrocellulose membranes (GE Healthcare Life Sciences, Uppsala, Sweden). The membranes were incubated for 1 h at room temperature in a blocking buffer (20 mM Tris–HCl, 137 mM NaCl, pH 8.0, containing 0.1 % Tween and 3 % skimmed dry milk), and probed with antibodies against Beclin, LC-3B and GAPDH (Cell Signaling, Beverly, MA, USA). The blots were developed with an HRP-conjugated secondary antibody and reacted proteins were visualized using an electrochemiluminescence (ECL) system (GE Healthcare Life Sciences).

## Results

### High-dose glucosamine induces pathological characteristics of OA

**Fig. 1:**
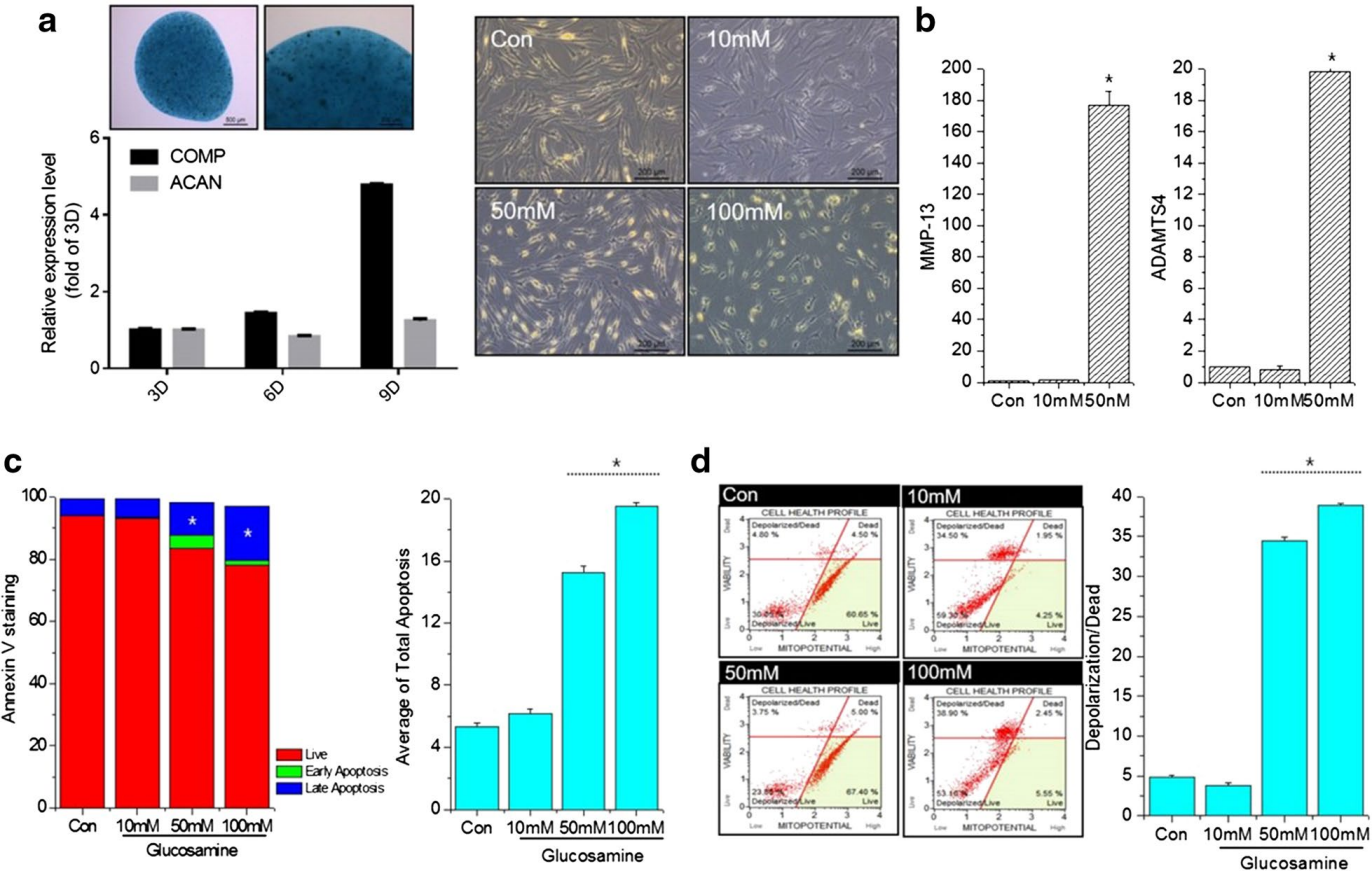
Effect of glucosamine in human normal chondrocytes. **a** Normal chondrocytes were cultured as a pellet and stained with Alcian blue at day 9 and the expression levels of COMP and ACAN were analyzed by qRT-PCR to check chondrogenic characteristics (*left panel*). Normal human chondrocytes were left untreated or were treated with 10, 50 or 100 mM glucosamine for 24  h. A dose-dependent change in the cell morphology was detected by phase-contrast microscopy (*right panel*). **b** RNA levels of MMP-13 and ADAMTS4 were analyzed by qRT-PCR. Cells were analyzed by **c** Muse™ Annexin V and Dead Cell assay and **d** Muse™ Mitopotential assay. *Bars* show the mean ± SD of three individual experiments. *P < 0.05 versus control (untreated)

Human articular chondrocytes were cultured in growth media and their chondrogenic capacity analyzed. Pellet culture of human chondrocytes showed an increase in Alcian blue staining and the transcription of a well-known cartilage matrix, cartilage oligomeric matrix protein (COMP) was increased as culture progressed. As previously mentioned [17–20], there is a significant controversial opinion on glucosamine effect, particularly with high dose of glucosamine. To verify the role of high-dose glucosamine in OA pathogenesis, chondrocytes were treated with increasing doses of glucosamine (10–100 mM). The number of chondrocytes was presenting severely degenerative morphology under glucosamine treatment and this degeneration was worsening in a dose-dependent manner (Fig. 1a). Consistent with morphological changes, the RNA levels of MMP-13 and ADAMTS4 were significantly increased in 50 mM-treated chondrocytes (Fig. 1b). To investigate the involvement of chondrocyte apoptosis in high-dose treatment of glucosamine, cells were stained with annexin-V/PI and analyzed by flow cytometry. After treatment with glucosamine for 24 h, the population of cells indicated a shift from viable cells to early and late stage apoptosis in a dose-dependent manner (Fig. 1c). The average percentage of late apoptotic cell populations increased to 15.25 and 19.54 %, respectively, for cells treated with 50 or 100 mM glucosamine suggesting that high dose of glucosamine may trigger chondrocyte apoptosis. Since mitochondrial dysfunction is closely related to cell death, we further assessed alterations in mitochondria potential (Fig. 1d). The average percentage of depolarized dead cells increased to 34.5 and 38.9 %, respectively, for cells treated with 50 or 100 mM glucosamine.

### High-dose glucosamine induced peroxisome dysfunction and accumulation of very long chain fatty acids (VLCFA)

**Fig. 2:**
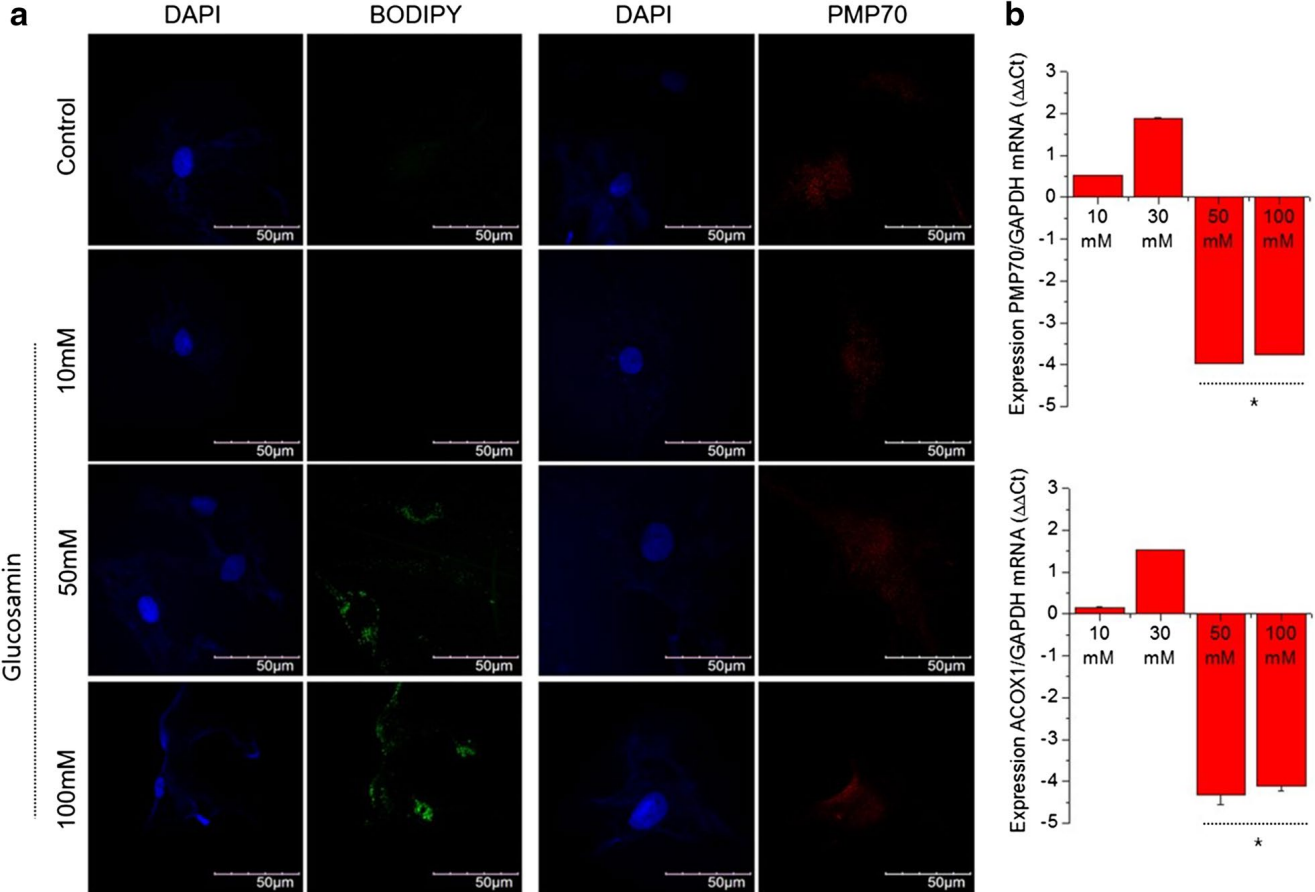
Effect of glucosamine on peroxisomal function. Normal human chondrocytes were left untreated or were treated with 10, 50, or 100 mM glucosamine for 24 h. **a** Cells were stained with BODIPY 493/503 and PMP70. **b** The RNA levels of PMP70 and ACOX1 were analyzed by qRT-PCR. *P < 0.05 versus control (untreated)

Recently, our laboratory suggested an interconnection between mitochondria and peroxisome during OA pathogenesis [20]. To investigate whether high-dose glucosamine induced peroxisomal dysfunction as well as mitochondrial dysfunction, normal chondrocytes were stained with BODIPY (493/503). We found that significantly large amounts of lipid were accumulated in cells treated with 50 and 100 mM glucosamine (Fig. 2a, left panel), indicating a possibility that high-dose glucosamine may induce dysfunction of peroxisome. To evaluate peroxisomal function, we determined the expression levels of PMP70/ABCD3, a70 kDa peroxisomal membrane protein and acyl-CoA oxidase 1 (ACOX1), which catalyzes the first step of peroxisomal fatty acid β-oxidation by qRT-PCR (Fig. 2b). The expression levels of PMP70 and ACOX1 were significantly decreased in cells treated with either 50 or 100 mM glucosamine.

**Fig. 3:**
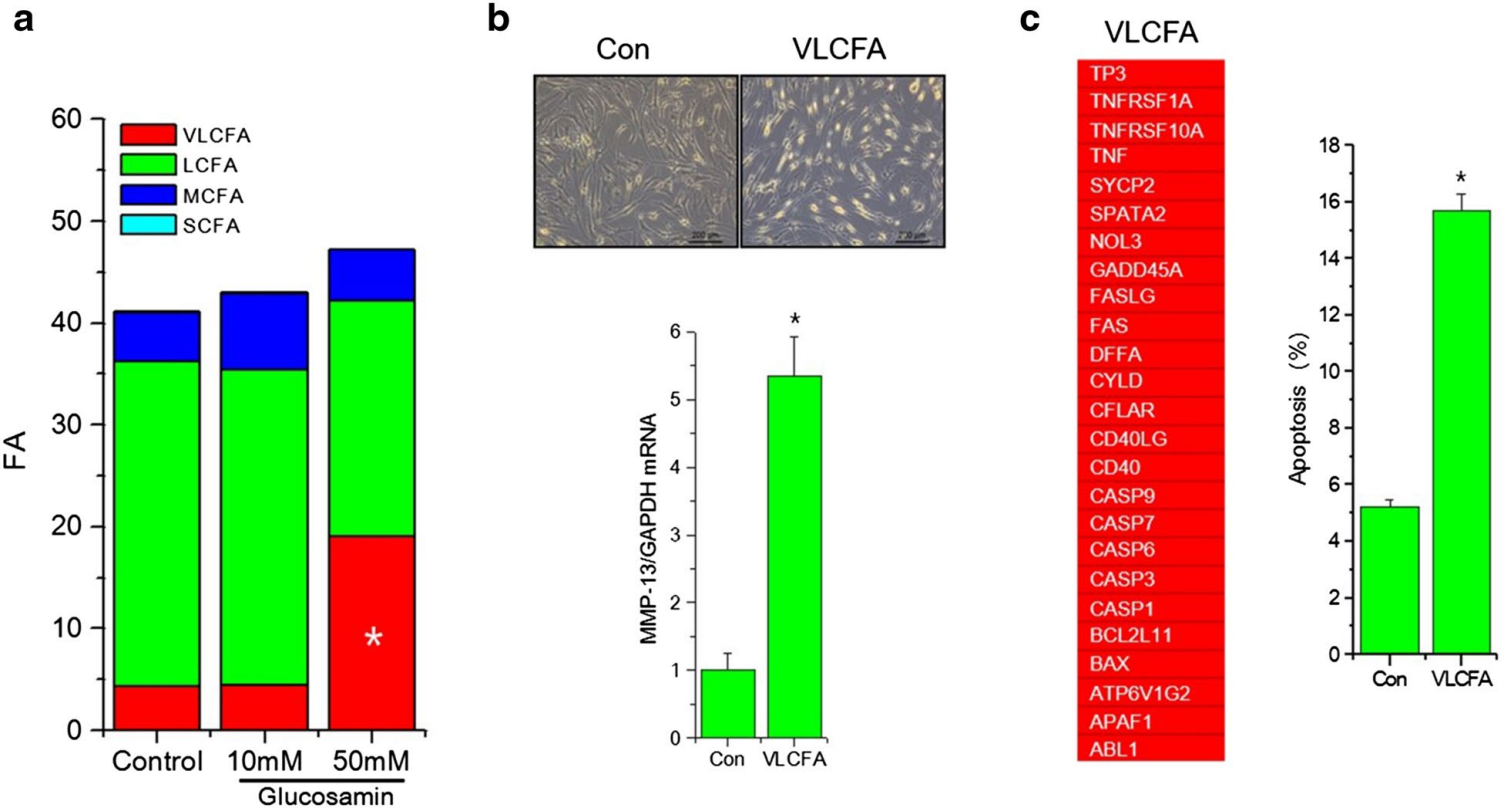
Effect of glucosamine on VLCFA. **a** Normal human chondrocytes were left untreated or were treated with 10 and 50 mM glucosamine for 24  h. Total lipid was analyzed using gas chromatography/mass spectrometry and divided into LLCFA, LCFA, MCFA, and SCFA. **b** Normal human chondrocytes were left untreated or were treated with lignoceric acid (VLCFA) for 24 h and cell morphologies were assessed by phase-contrast microscopy (*upper panel*). RNA levels of MMP-13 were analyzed by qRT-PCR (*lower panel*). **c** RNA levels of pro-apoptotic genes were analyzed by qRT-PCR and presented as heat-map. *Red* color represented as significant increase in expression levels. Apoptotic cell populations were analyzed by FACS. *Bars* show the mean ± SD of three individual experiments. *P < 0.05 versus control (untreated)

Next, we investigates the possibility that this peroxisomal dysfunction by glucosamine could be resulted in lipid accumulation due to lipid metabolism impairment. Lipidomics analysis (Table 1) showed that treatment with 50 mM glucosamine lead to the accumulation of very long chain fatty acids (VLCFA) in normal chondrocytes (Fig. 3a). By adding exogenous lignoceric acid to the cells, we evaluated the effects of VLCFA on lipid metabolism. Cells treated with VLCFA showed a severely degenerative morphology (Fig. 3b, upper panel). Furthermore, cells treated with lignoceric acid demonstrated significantly increased MMP-13 RNA levels (Fig. 3b, lower panel). Moreover, levels of pro-apoptotic genes including FASL, FAS and BAX, as well as the percentage of apoptotic cell populations, were increased with lignoceric acid treatment (Fig. 3c). These data suggest that high-dose glucosamine-induced VLCFA accumulation may be responsible for chondrocyte apoptosis and MMP-13 induction.

**Table 1:** Lipidomics of glucosamine-treated chondrocytes

Control		Glucosamine 10 mM		Glucosamine 50 mM	
Name	%	Name	%	Name	%
Nonahexacontanoic acid	0.07	Nonahexacontanoic acid	0.042		0.125
Tetratetracontane	0.21	Glycerol tristearate	0.518	Nonahexacontanoic acid	0.17
Hentriacontane	0.04	Cholesta-3,5-diene	0.304	Benzenepropanoic acid,3,5-bis(1,1-dimethylethyl)-4-hydroxy-	0.197
Cholesta-3,5-diene	0.12	2,4,6-Triphenyl-1-hexene-D5	3.155	Cholesta-3,5-diene	0.124
Hexacosane	0.07	Docosane	0.393	Tetracosane	2.598
2,4,6-Triphenyl-1-hexene	3.09	rac Methadone *N*-oxide	0.288	Dioctyl terephthalate	2.483
tricosane	0.08	Eicosane	5.42	2,4,6-Triphenyl-1-hexene-D5	0.28
Docosane	0.5	Eicosanoic acid	0.849	Docosane	13.003
*cis*-4,7,10,13,16,19-Docosahexaenoic acid	0.14	Methyl arachidonate	0.122	Erucylamide	0.612
Octadecanoic acid 2,3-dihydroxypropyl ester	0.59	(S)-(E)-(-)-4-acetoxy-1-phenyl-2-dodecen-1-one	0.046	Glycerol b-monostearate	0.06
Nonadecyl trifluoroacetate	0.06	1-Nonadecanol	0.196	1-Dodecanol, 2-octyl- eicosane	1.733
Eicosane	3.32	2-Hexadecanoyl glycerol	0.877	5,8,11,14,17-Eicosatetraenoic acid	0.152
Eicosanoic acid	0.83	Methyl linoleate	0.112	Nonadecane	0.08
Arachidonic acid	0.41	Octadecanoic acid	1.746	2-Hexadecanoyl glycerol	1.397
5,8,11,14,17-Eicosapentaenoic acid	0.09	1-Octadecene	0.115	1-Nonadecene	0.094
Hexadecanoic acid,2-hydroxy-1-(hydroxymethyl) ethyl ester	1.39	9-Octadecenamide, (Z)-	4.827	Octadecanoic acid	1.556
Methyl 9-(Z)-octadecenoate	1.12	11-Octadecenoic acid,(11Z)-	0.094	9-Octadecenamide, (Z)-	0.91
9,12-Octadecadienoic acid (Z,Z)-, methyl ester	0.31	Palmitic acid	1.343	Linoleic acid	0.141
Thiocarbamic acid	0.28	2-Propenoic caid, tridecyl ester	0.465	11-Octadecenoic acid,(11Z)-	0.386
Octadecane	0.15	Hypogeic acid	0.35	*trans*-9-octadecenoic acid	0.091
Stearic acid	2.21	[2.2]Paracyclophane	0.059	Pentafluoropropionic acid tetradecyl ester	0.136
1-Octadecene	0.11	3-Methyl-2-benzylindole	4.12	2-Ethylhexyl methyl isophthalate	0.065
9-Octadecenamide, (Z)-	5.98	2,4-Diphenyl-1-butene-D5	0.437	1-Decanol,2-hexyl-	0.059
Heptafluorobutyric acid, *n*-tetradecyl ester	0.18	1-Pentadecene	0.129	Hexadecane	0.142
Methyl hexadecanoate	1.68	2-Methyl-1-anthracenamine	6.432	Palmitic acid	1.374
Cyclopentolate	0.14	1-Methyl-2-anthracenamine	2.094	Benzene,1,1′-(1,2-cyclobutanediyl)bis-, *trans*-	0.28
Fumaric acid, 2-ethylhexyl hexyl ester	0.11	Tetradecane	0.125	3-Methyl-2-benzylindole	5.108
Hexadecane	0.07	2-Tetradecene	0.162	2,4-Diphenyl-1-butene-D5	0.337
Hexadecanoic acid	0.33	Phenol,2,5-bis(1,1-dimethylethyl)-	0.079	1,3-diphenyl-cyclobutane	0.06
2-Propenoic acid, tridecyl ester	1.41	5-Methoxy-2-methyl-1-propyl-1H-indole-3-carboxylic acid	0.451	Propanoic acid, 3-mercapto-, dodecyl ester	0.234
2,4-Diphenyl-1-butene	0.47	Dodecahydropyrido[1,2-b]isoquinolin-6-one	0.053	2-Methyl-1-anthracenamine	5.093
Benzene,1,1′-(1,2-cyclobutanediyl)bis-, *cis*-	0.37	Sulfurous acid, dodecyl 2-propyl ester	0.155	1H-Indole,2-methyl-3-phenyl-	1.934
3-Methyl-2-benzylindole	4.11	Dodecane	0.115	2-Ethylacridin	0.553
Cyclobutane,1,3-diphenyl-,*trans*	0.06	Cyclododecane	0.12	Benzo[h]quinoline,2,4-dimethyl-	0.113
Propanoic acid,3-mercapto-, dodecyl ester	0.66	1-Methyl-5,6-dimethoxy-2,3-dihydroindole	2.373	Tridecane, 6-methyl-	0.164
Benzene,1,1′-(1,3-propanediyl)bis-	0.07	2,7-Naphthalenedisulfonicacid, 3-hydroxy-4-nitroso-	0.37	Tetradecane	0.084
1-Methyl-2-anthracenamine	4.45	1-Decene	0.191	2,4-Di-tert-butylphenol	0.094
1-Tetradecanol	0.24	2-Methylmercaptobenzothiazole	0.498	Benzene,1,3-bis(1,1-dimethylethyl)-	0.152
Tetradecane	0.12	1,4-Benzenedicarboxylic acid	3.412	12-Hydroxydodecanoic acid	0.224
2,4-Di-tert-butylphenol	0.13	Benzene	0.19	2-Propenoic acid oxybis(methyl-2,1-ethanediyl) ester	0.699
2-Tridecanone	0.17	Fumaric acid	0.08	Ethyl 4-etoxybenzoate	0.074
Dodecahydropyrido[1,2-b]isoquinolin-6-one	0.06	2-Chloropropionic acid	0.069	Cycl zodecane	0.249
Decane, 2, 3, 5-trimethyl	0.16			exo-5-hydroxy-exo-2,3,3a,4,5,6,7,7a-octahydro-4,7-methano-1H-indene	0.079
1-Dodecanol	0.1			S,S′-Bis(2-dimethylaminoethyl) methylphosphonodithioate	0.063
2-Carbomethoxy-1,2,3,4-tetrahydronaphthalene	0.09				
2-Undecanone	0.11				
4-Ethoxybenzoic acid ethyl ester	0.11				
5-Acetyl-2-methoxyphenyl-1-thiocyanate	0.55				
3,5-Dimethylbenzaldehyde thiocarbamoylhydrazone	0.05				
2-Methylmercaptobenzothiazole	0.16				
Terephthalic acid	3.32				
octanoic acid	0.09				
2-Xylene-D10	0.11				
Benzene	0.14				
Carbonochloridic acid, decyl ester	0.16

### High-dose glucosamine stimulates autophagic cell death

**Fig. 4:**
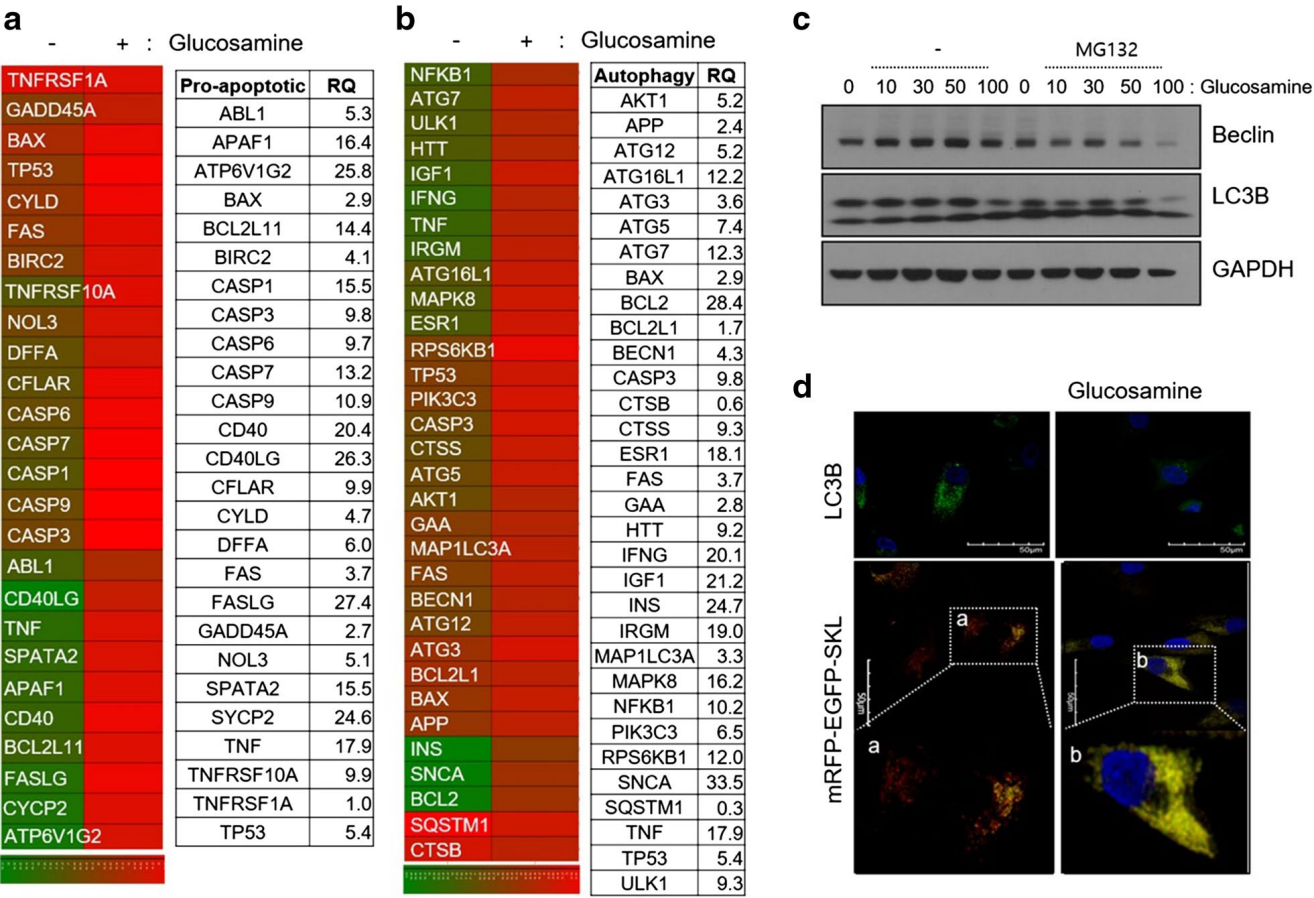
Effect of glucosamine on autophagy in human normal chondrocytes. Normal human chondrocytes were treated with 50 mM glucosamine and the RNA levels of **a** pro-apoptotic genes and **b** autophagy-related genes were analyzed by qRT-PCR and expressed as a heat-map and value of relative quantity (RQ). **c** Normal human chondrocytes were treated with 10, 50, or 100 mM glucosamine in the absence or presence of MG132 and the dose-dependent change in protein levels of beclin and LC3B was detected by immunoblotting. GAPDH was used as a loading control. **d** Normal human chondrocytes were treated with 50 mM glucosamine and pexophagy was examined by introducing an mRFP-EGFR-SKL construct. Results shown are representative of at least four independent experiments. *P < 0.05 versus control (untreated)

To evaluate whether autophagic cell death is involved in glucosamine-induced apoptosis, the expression levels of genes involved in apoptosis (Fig. 4a) and autophagy (Fig. 4b) were analyzed. Both levels of apoptotic genes and autophagic genes assessed here were significantly increased to more than threefold in cells treated with 50 mM glucosamine. RNA levels of caspase-1, -3, -6, -7, and -9 demonstrated a 15.5-, 9.8-, 9.7-, 13.2-, and 10.9-fold increase, respectively (Fig. 4a). RNA levels of ATG3, ATG5, ATG7, ATG12, and ATG16L1 showed a 3.6-, 7.4-, 12.3-, 5.2-, and 12.2-fold increase, respectively (Fig. 4b). Moreover, expression levels of beclin-1 were increased 4.3-fold in cells treated with 50 mM glucosamine. To confirm that glucosamine induced the hyper-activation of autophagic response, normal chondrocytes were treated with glucosamine in the absence or presence of MG132, a cell-permeable proteasome inhibitor. In the presence of MG132, the cells exposed to glucosamine showed slightly decreased levels of autophagy markers becin-1 and LC-3B, suggesting that the treatment with high-dose glucosamine may actually decrease autophagic responses (Fig. 4c). In addition, the pattern of microtubule-associated LC3, as assessed by immunostaining, indicated inactivation of autophagic response under glucosamine treatment (Fig. 4d, upper panel). Next, we used the tandem fluorochrome pexophagy assay with mRFP-EGFP-SKL as a reporter. Cells treated with glucosamine did not display the characteristic “red only” structure as a consequence of EGFP fluorescence quenching by acid pH after the delivery of peroxisome to lysosome, which would indicate the stimulation of autophagic responses (Fig. 4d, lower panel).

**Fig. 5:**
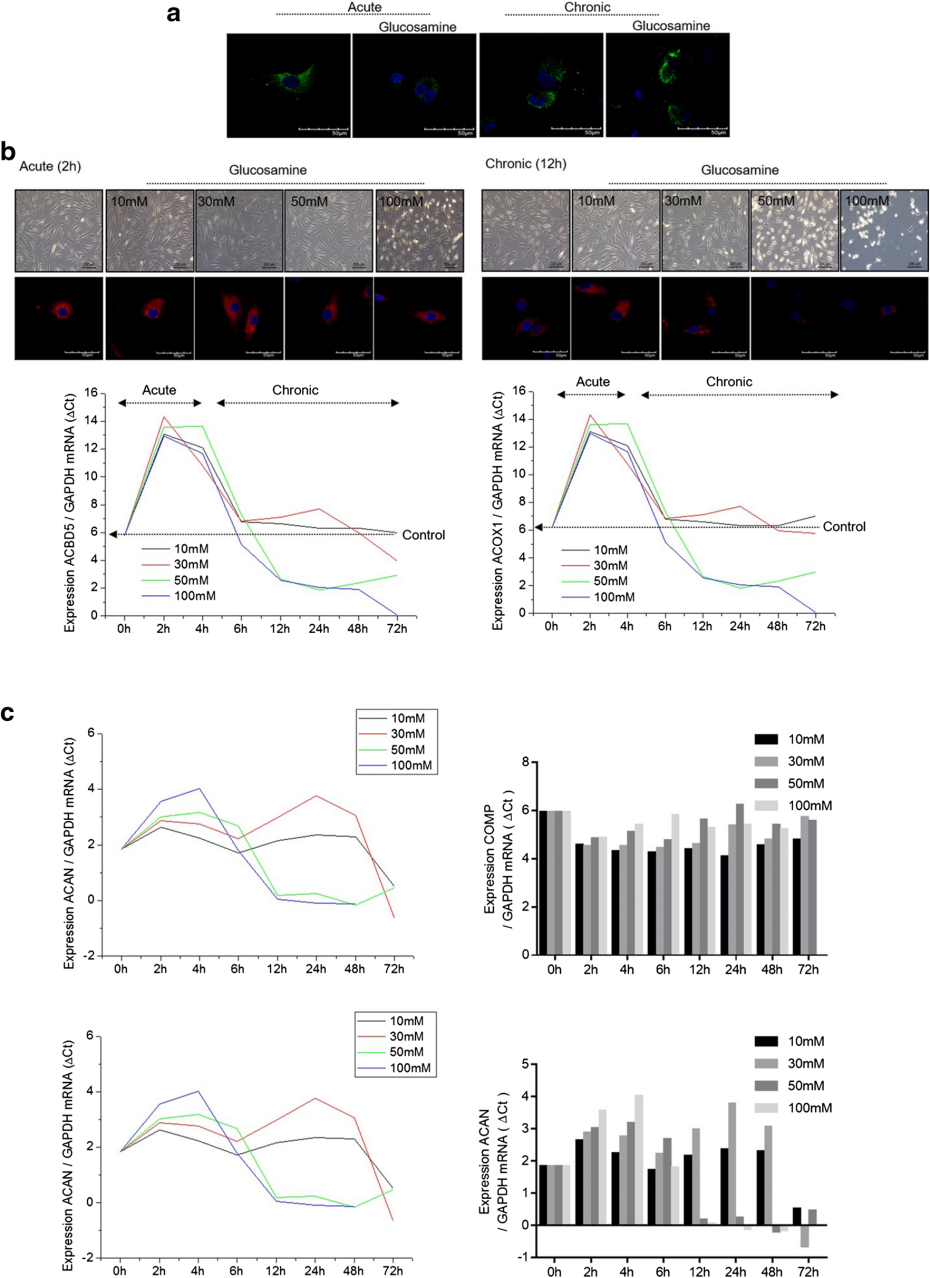
Effect of short time exposure vs. long time exposure of glucosamine. **a** Normal human chondrocytes were treated with 50 mM glucosamine for 2 or 24 h and the autophagosome formation was analyzed by introducing LC3-GFP constructs. **b** Normal human chondrocytes were left untreated or were treated with 10, 30, 50, or 100 mM glucosamine for 2 or 24 h. Cell morphology was detected by phase-contrast microscopy and peroxidation was analyzed by BODIPY 665/676 staining (*upper panel*). The RNA levels of ACBD5 and ACOX1 were analyzed by qRT-PCR (*lower panel*). Results shown are representative of at least four independent experiments. **c** RNA levels of COMP and ACAN were analyzed by qRT-PCR. Results shown are representative of at least four independent experiments

**Fig. 6:**
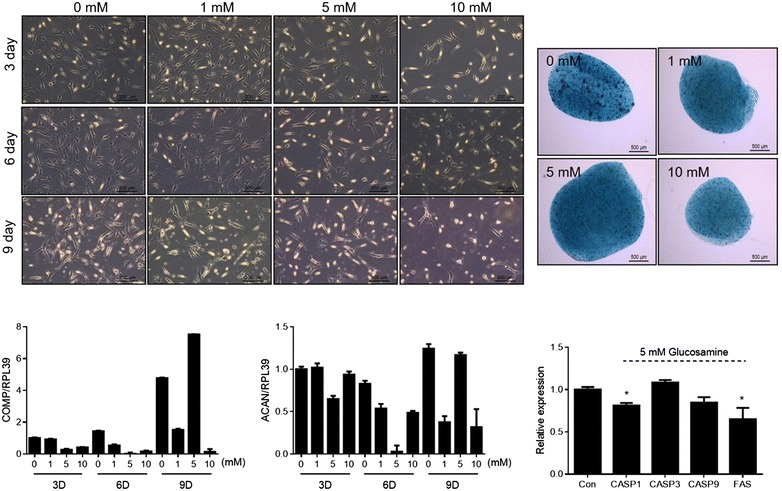
Effect of short time exposure vs. long time exposure of glucosamine. Normal human chondrocytes were cultured as monolayer or pellet and treated with 1, 3, or 10 mM glucosamine for 9 days. Cell morphology of monolayer culture was detected by phase-contrast microscopy and pellet cultures were stained with Alcian blue at day 9 (*upper panel*). The expression levels of cartilage matrix genes such as COMP, ACAN and apoptotic genes such as CASP1, 3, 9, FAS were analyzed by qRT-PCR. Results shown are representative of at least four independent experiments. *P < 0.05 versus control (untreated)

As mentioned earlier, the effect of glucosamine in OA is still controversial. Whilst some reports showed that high-dose glucosamine may stop the degradation of cartilage by acting as a chondro-protective agent [21], others have reported a negative effect of high-dose glucosamine during OA pathogenesis [22], suggesting the possibility that the effects of glucosamine may depend on treatment length or duration. Therefore, we analyzed microscopy-based GFP-LC3 puncta formation in cells exposed to 50 mM glucosamine, to address this possibility. At exposure times of 2 h (short time exposure), glucosamine was found to induce GFP-LC3 puncta. However, glucosamine significantly suppressed GFP-LC3 puncta at 24 h exposure (Fig. 5a). Next, we analyzed whether the difference in the effects of glucosamine based on the exposure time (short time exposure vs. long time exposure) is a result of peroxisomal dysfunction. Peroxisomal oxidation was not affected during short time exposure of glucosamine, although during long time exposure of glucosamine, we observed a significant reduction in peroxisomal oxidation (Fig. 5b). Consistent with these findings, the expression levels of acyl-CoA-binding protein (ACBD5), a typical marker for pexophagy, or acyl-CoA oxidase 1 (Acox-1), an initial enzyme in β-oxidation, increased during short time exposure of glucosamine but decreased during long time exposure. A typical cartilage matrix gene, COMP was dramatically decreased during short time exposure (Fig. 5c). Moreover, when normal chondrocyte were exposed with low concentration, we found that 5 mM glucosamine increased transcription of cartilage matrix gene, COMP and suppressed apoptotic genes such as CASP1 and FAS (Fig. 6).

## Discussion

Glucosamine is the main component of proteoglycans, and the major non-collagenous cartilage-specific proteoglycan of the intervertebral disc is aggrecan. In the clinic, glucosamine has been used in the treatment of OA, although the application has not been widely accepted and remains controversial [7, 8]. In this study, we demonstrated that glucosamine could act differently depending on its dosage and period of treatment. Exposure of chondrocytes to high-dose glucosamine induced lipid accumulation, possibly through suppression of PMP70 and ACOX1, and resulted in the stimulation of cell death, thus indicating a regulatory role for glucosamine in peroxisomal function. Since the peroxisome is involved in lipid metabolism, including β-oxidation of fatty acids and transfer of oxidized lipid to mitochondria for utilization in energy metabolism [23–25], peroxisomal dysfunction could be a trigger for OA-induced chondrocyte death.

One of the typical characteristics in OA pathogenesis is chondrocyte apoptosis in cartilage, but the underlying regulatory mechanisms have not been well studied. Recently, nitric oxide (NO) and reactive oxygen species (ROS) have been suggested to be key factors in mediating chondrocyte apoptosis through mitochondrial dysfunction such as damage of mitochondrial DNA [26]. Impairment of mitochondrial events have been known to be involved in chondrocyte apoptosis, including reduction of the mitochondrial transmembrane protein complex IV, decrease of mitochondrial membrane potentials and release of cytochrome c [27]. In the present report, we observed that long-term exposure to glucosamine induced impairment of mitochondrial membrane potentials, suggesting the involvement of glucosamine in the regulation of mitochondrial function during OA pathogenesis.

Recently, other and our group have provided evidence for an interconnection between cellular organelles, particularly between the mitochondria and the peroxisome. Peroxisome dysfunction hindered mitochondrial function and vice versa. Likewise, we also found in this study that glucosamine induced dysfunction of mitochondria as well as that of the peroxisome in human chondrocytes. Long-term exposure to glucosamine induced the accumulation of VLCFA and this could be responsible for chondrocyte apoptosis. Recent studies showed a correlation between VLCFA accumulation and cell death as a result of the silencing of peroxisomal transporters and altered mitochondrial function [28]. ABCD-1 deficiency induced apoptosis in oligodendrocytes and led to astrocytic inflammatory responses and loss of myelin [29, 30]. In addition, VLCFA depletion is known to result in constitutive activation of autophagy [31], indicating that VLCFAs serve to dampen the amplitude of autophagy. VLCFA accumulation induced by long-term exposure to glucosamine could be responsible for suppression of autophagic responses in OA chondrocytes. This suggests that the functional integrity of peroxisomes in the regulation of VLCFA has important implications for autophagy and cell homeostasis.

Further, we described, for the first time, a direct correlation between glucosamine and pexophagy. Even though glucosamine is widely used to treat or prevent OA in humans, the effects of treatment on cartilage degeneration are controversial. We showed that glucosamine exhibits a concentration- and exposure time-dependent dual action in the regulation of chondrocyte survival and apoptosis. Recent research on an association between glucosamine and the PI3K/AKT/mTOR signaling pathway has elicited controversial reports. Caramés et al. had demonstrated that the mTOR pathway was inhibited by glucosamine in chondrocytes [15]. In contrast, Shintani et al. found that the mTOR pathway did not participate in glucosamine-induced autophagy in HeLa and COS7 cells [32]. Here, short exposure of glucosamine activated autophagic responses (referred to as short time response), whereas long exposure of glucosamine inhibited autophagic response, particularly pexophagy and led to impairment of peroxidation (referred to as long time response). Taken together, these results indicate that depending on the period of exposure, glucosamine may act as both a suppressor and inducer of apoptosis and autophagy.

## Conclusions

Collectively, our data demonstrated the role of glucosamine in autophagy and showed a direct correlation between glucosamine and peroxisomal function. We also highlighted a dual role of glucosamine in apoptosis and autophagy in human chondrocytes. Glucosamine exerts exposure time-dependent dual effects on apoptosis and autophagy. Although further research is required to fully understand the relationship between dosage and exposure time to glucosamine, the differential opposing effects of glucosamine indicate that dosage and duration of treatment may be a determining factor in the line of beneficial reward-to-risk action in OA therapy.
