# Erratum to: Beneficial reward-to-risk action of glucosamine during pathogenesis of osteoarthritis

**DOI:** 10.1186/s40001-016-0197-x

**Published:** 2016-01-29

**Authors:** Yeon-Ho Kang, Sujeong Park, Chihyun Ahn, Jinsoo Song, Dongkyun Kim, Eun-Jung Jin

**Affiliations:** Department of Biological Sciences, College of Natural Sciences, Wonkwang University, Iksan, Chunbuk 570-749 Korea; Integrated Omics Institute, Wonkwang University, Iksan, Chunbuk 570-749 Korea

## Erratum to: Eur J Med Res (2015) 20:89 DOI 10.1186/s40001-015-0176-7

After publication of the original article [1], the authors noticed the Acknowledgements section was incorrect as follows: “This works was supported by National Research Foundation (NRF) of Korea Grant funded by the Korean Governments by the Korea government (MSIP) [2013R1A1A2011999], [NRF-2013R1A2A2A01067194], and [2011-0030130]. The funders had no role in study design, data collection and analysis, decision to publish, or preparation of the manuscript.”

The correct version of the Acknowledgements section is included below:
